# Case Report: Sustained complete remission on combination therapy with olaparib and pembrolizumab in BRCA2-mutated and PD-L1-positive metastatic cholangiocarcinoma after platinum derivate

**DOI:** 10.3389/fonc.2022.933943

**Published:** 2022-07-25

**Authors:** Taotao Zhou, Robert Mahn, Christian Möhring, Farsaneh Sadeghlar, Carsten Meyer, Marieta Toma, Barbara Kreppel, Markus Essler, Tim Glowka, Hanno Matthaei, Jörg C. Kalff, Christian P. Strassburg, Maria A. Gonzalez-Carmona

**Affiliations:** ^1^ Department of Internal Medicine I, University Hospital of Bonn, Bonn, Germany; ^2^ Department of Radiology, University Hospital of Bonn, Bonn, Germany; ^3^ Department of Pathology, University Hospital of Bonn, Bonn, Germany; ^4^ Department of Nuclear Medicine, University Hospital of Bonn, Bonn, Germany; ^5^ Department of Visceral Surgery, University Hospital of Bonn, Bonn, Germany

**Keywords:** cholangiocarcinoma, BRCA2, PDL1, targeted therapy, case report

## Abstract

Cholangiocarcinoma (CCA) still has a poor prognosis and remains a major therapeutic challenge. When curative resection is not possible, palliative systemic chemotherapy with gemcitabine and platinum derivate as first line followed by a 5-FU doublet combination as second line is the standard therapy. Recently, targeted therapy and immunotherapy have rapidly emerged as personalized therapeutic approaches requiring previous tumor sequencing and molecular profiling. BRCA mutations are well-characterized targets for poly (ADP-ribose) polymerase inhibitors (PARPi). However, BRCA gene mutations in CCA are rare and few data of PARPi in the treatment of CCA are available. Immunotherapy with programmed death receptor-1 (PD-1) has been shown to be effective in combination with chemotherapy or in PD-L1-positive CCA. However, data from immunotherapy combined with targeted therapy, including PARPi, are lacking. In this report, we present the case of a male patient with PD-L1-positive and BRCA2-mutated metastatic intrahepatic cholangiocarcinoma, who was treated with a combined therapy with PARP (PARPi), olaparib, and a PD-1 antibody, pembrolizumab, as second-line therapy after gemcitabine/platinum derivate failure. Combined therapy was able to induce a long-lasting complete remission for over 15 months. The combined therapy was feasible and well tolerated. Only mild anemia and immune-related thyroiditis were observed, which were easily manageable and did not result in discontinuation of olaparib and pembrolizumab.

**Conclusion:**

The presented case showed substantial clinical activity of a combination with olaparib/pembrolizumab in advanced BRCA2-mutated CCA. Thus, identifying targetable molecular signatures and combinations of targeted therapies with immunotherapy reveals a promising strategy to effectively treat patients with cholangiocarcinoma and should be considered after failure of standard chemotherapy.

## Introduction

Cholangiocarcinoma (CCA) is a comparably rare, albeit highly aggressive tumor entity. It is reported to represent 3% of malignant gastrointestinal diseases (1, 2). Its incidence, especially in the case of intrahepatic CCA, seems to be rising due to changes in classification systems and improvement in diagnostic methods (3, 4). Since clinical presentation in its early stage is usually unspecific and adequate preventive screening methods are lacking, in most cases, cholangiocarcinoma is diagnosed in the non-curative state.

Cholangiocarcinoma is usually distinguished by anatomical localization and categorized as intrahepatic (iCCA), perihilar (pCCA), or distal (dCCA). This classification is clinically highly relevant as pathogenesis, clinical presentation prognosis, and molecular profile differ distinctly between these subgroups (5–8).

Even with oncological resection and following adjuvant therapy, the rate of recurrence might be up to 50%–70% within the first 2 years (9, 10) and 5-year overall survival ranges between 25% and 40% (11).

In metastatic or irresectable cases, palliative chemotherapy, mostly with gemcitabine in combination with platinum derivate, is the first-choice treatment based on the findings of the pivotal randomized phase III trial ABC-02. Further local therapies, such as radiation therapy (12, 13), endobiliary therapy (14, 15), or chemoembolization (16–18), can be applied individually or additionally in a multimodal approach (19). However, 5-year survival is still estimated with 5%–15% (10). Few further studies have identified any significant effect of systemic chemotherapy as second-line treatments (20, 21). However, emerging evidence has shown that CCA is associated with several genetic alterations, including mutations or fusions, that can be actionable by targeted therapy and which have been demonstrated to be effective in CCA in phase II and III trials (22). Hence, screening for molecular mutations in tumor tissue has fundamentally gained relevance and might even be considered obligatory. Combinations of immune checkpoint inhibitors (ICI) with chemotherapy or targeted therapy have been shown to be more effective than monotherapy in several tumor entities. For CCA, the combination of gemcitabine/cisplatin with PD-L1 (programmed cell death ligand 1) antibody, durvalumab, has recently shown a superior antitumor activity than standard chemotherapy in a phase III trial (TOPAZ-1) (23). Regarding combinations of ICI and targeted therapy, there is no evidence from clinical trials to date for advanced cholangiocarcinoma.

Here, we report on a case of a patient with PD-L1-positive and BRCA2-mutated metastatic intrahepatic cholangiocarcinoma who was treated with the PARP inhibitor olaparib and the PD-1 inhibitor pembrolizumab as second-line therapy after gemcitabine and platinum derivate and who achieved sustainable and complete remission.

## Case description

In August 2017, a 53-year-old Caucasian man was referred to the university hospital of Bonn, Germany, with elevated liver transaminases and cholestasis parameters after cholecystectomy in March 2017, which was performed due to cholecystolithiasis. The patient had a known medical history of arterial hypertension as well as Hashimoto’s thyroiditis that did not require hormone substitution at that time.

**Figure 1:**
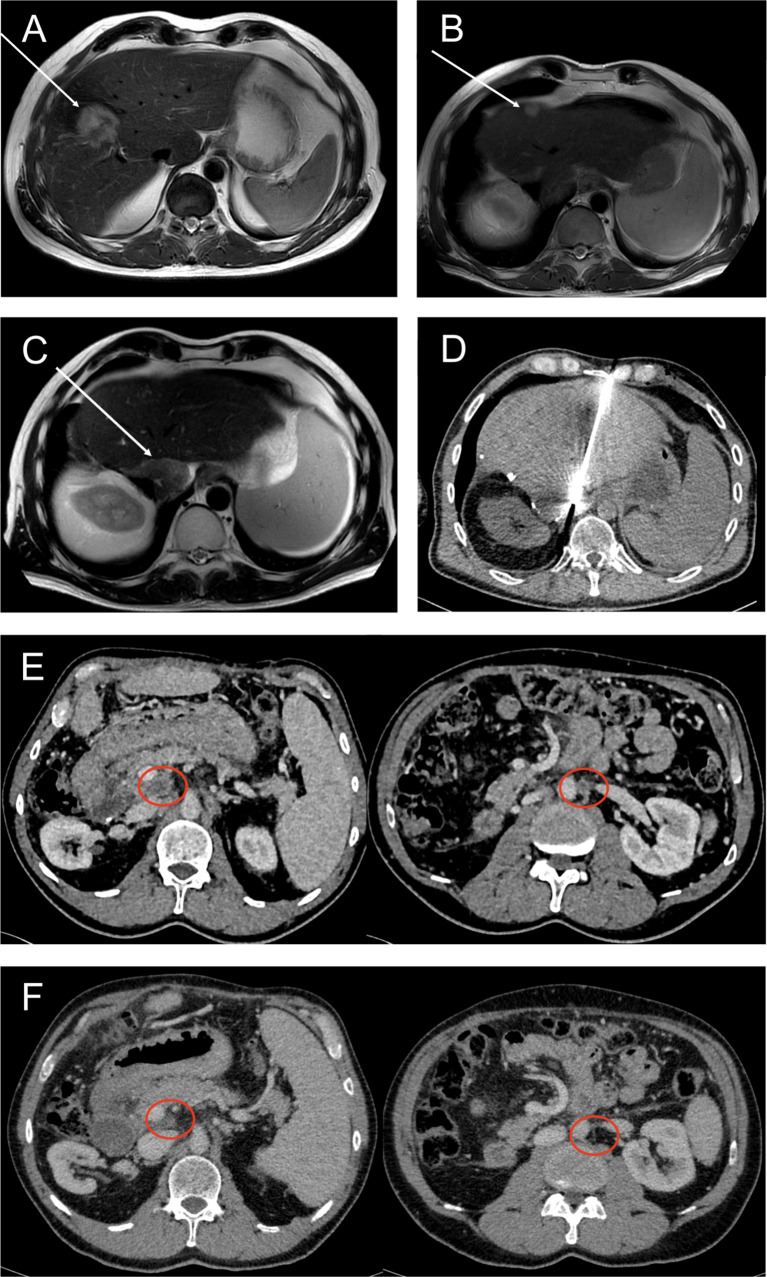
Representative CT and MRI scans showing different disease stages and local ablative therapy during the disease course. **(A)** MRI (T2) of July 2017. White arrow points toward intrahepatic cholangiocarcinoma in segment V/VIII at first presentation. **(B)** MRI (T2) of October 2018 revealing a singular hepatic metastasis (white arrow). **(C)** Second intrahepatic recurrence of CCA (white arrow) diagnosed by MRI (T2) in February 2020. **(D)** CT-guided microwave ablation of this second hepatic metastasis in February 2020. **(E)** CT (portal venous phase) showing central necrotic lymph node metastases (red circles) in October 2020, before initiating olaparib/pembrolizumab combination therapy. **(F)** In July 2021, CT scan (portal venous phase) shows partial and complete remission of lymph node metastases after about 9 months of treatment with olaparib and pembrolizumab.

**Figure 2:**
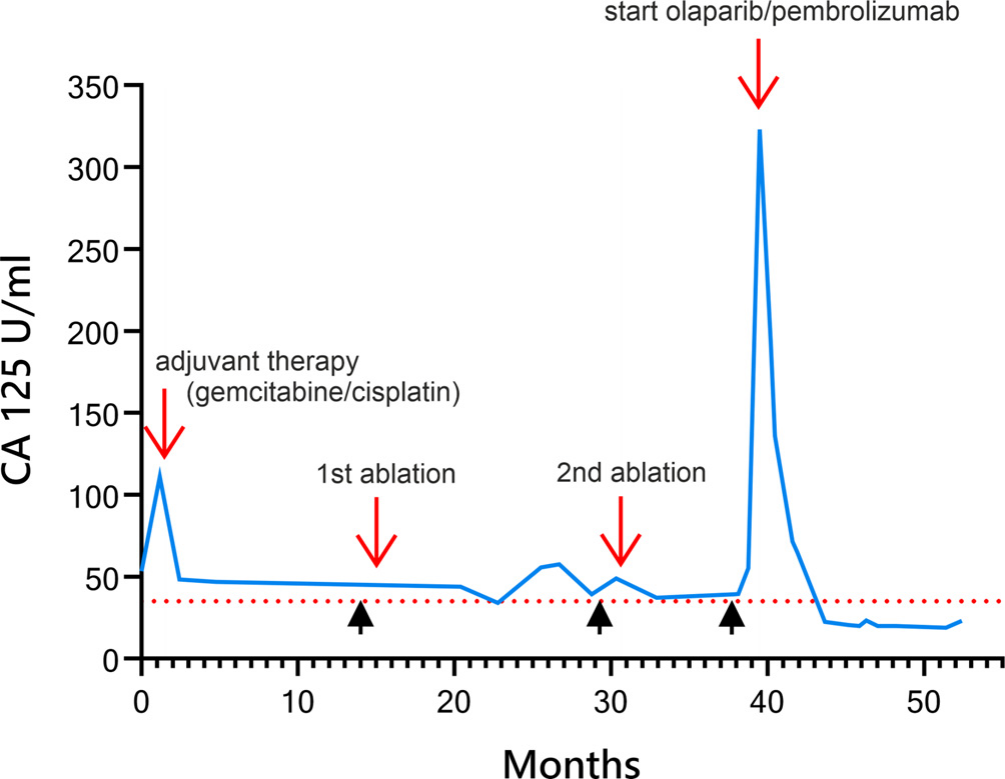
Diagram of development of Ca 125 in U/ml over time after primary hemihepatectomy during the disease course and under multimodal therapy. Red arrows are labeled with therapeutic events. Black arrow heads indicate time of recurrence as diagnosed by imaging.

CT as well as MRI scan revealed a 35 mm × 45 mm mass forming lesion in liver segment V/VIII without signs of thoracoabdominal metastasis (see **Figure 1A**). CEA (0.7 ng/ml) and Ca 19-9 (<2 U/ml) were low and remained within the normal range during the entire disease course, whereas Ca 125 was slightly elevated (53.3 U/ml, ULN 35 U/ml) (see **Figure 2**). A CT-guided liver biopsy revealed infiltrates of adenocarcinoma with strong CK7 positivity, consistent with cholangiocarcinoma. The PD-L1 status was negative. According to the decision of our interdisciplinary tumor board and after excluding extrahepatic tumor manifestations (colonoscopy, gastroscopy, CT scan), right hemihepatectomy with lymph node dissection and bile duct reconstruction *via* biliodigestive anastomosis was performed in August 2017. The examination of the resectate confirmed high-grade intrahepatic CCA with postoperative pT2, pN0, L1, V1, Pn1, and R1 stages. Ca 125 increased to 111 U/ml after surgery. Due to the R1 situation and the postoperative positive CA125, an additive therapy with gemcitabine/cisplatin was initiated in October 2017. Due to a grade 3 (CTCAE) polyneuropathy, cisplatin had to be discontinued in February 2018, after seven cycles. Since radiology imaging presented a complete remission at this time and initial resection revealed marginal involvement, a second-look exploration with follow-up resection of the former resection site was performed in March 2018 following the decision of our interdisciplinary tumor conference. A histological review of the suspected remaining carcinoma revealed no microscopic malignancy. Therefore, a close follow-up was initiated hereafter beginning in March 2018.

**Figure 3:**
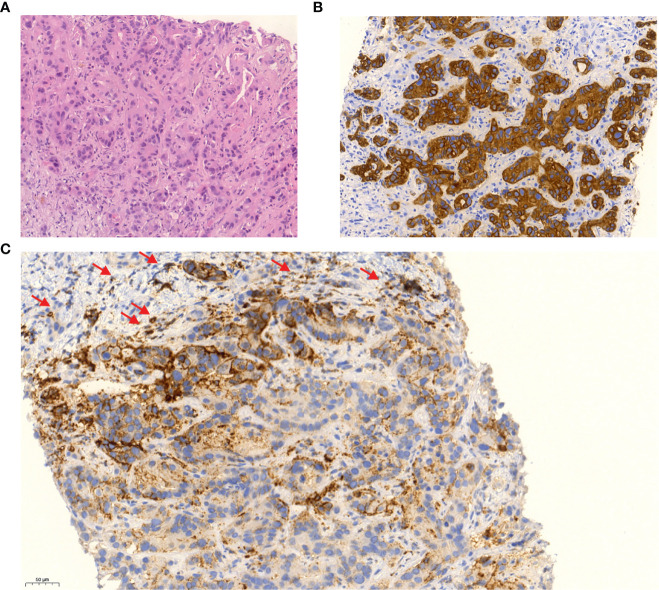
Liver biopsy (×20 magnification) of second hepatic metastasis in February 2020 shows infiltrates of atypical glandular proliferates in hematoxylin and eosin staining **(A)**, which were positive for CK7 **(B)**. Immunohistochemistry staining shows weak to moderate membranous expression of PDL1 in about 50% of the tumor cells and in about 20% of surrounding immune cells **(C)**.

In October 2018, after 6 months of stable remission, a solitary hepatic metastasis (see **Figure 1B**), histologically confirmed by biopsy (see **Figure 3**), was diagnosed. As systemic therapy was poorly tolerated and no other tumor manifestations were evident, we decided on local microwave ablation therapy with close follow-up. The patient remained in remission up to February 2020, when a second, singular hepatic metastasis was also treated with percutaneous microwave ablation therapy (see **Figures 1C, D**). During close follow-up examinations, CT of the abdomen revealed peritoneal metastasis with ascites as well as lymph node metastasis in November 2020 (see **Figure 1E**). Ca 125 reached 323 U/ml, consistent with radiological findings (see **Figure 2**).

Previously, during the past follow-up in June of 2019, cancer tissue was analyzed using next-generation sequencing (FoundationOne^®^ CDx) to identify possible molecular targetable alterations. One of the findings revealed an alteration in the BRCA2 gene (E1493fs*10). Further alterations were detected in the NF2 gene, APC gene, TP53 gene, CDKN2A gene, and MLL 2. The microsatellite status was stable. Tumor mutational burden was comparably low (4 mutations/Mb). There was no evidence of FGFR2 mutation or fusion, NTRK fusion, IDH1, IDH2, BRAF, or NRAS mutations.

Family history of the patient was positive in first-degree relatives for pancreatic carcinoma (father) and mammary carcinoma (sister). Therefore, germline analysis (TruRisk Panel^®^) of BRCA1 and -2 genes was performed upon revelation of BRCA2 mutation in tumor tissue, which showed a matching BRCA2 mutation in the germline.

Considering the well-known activity of platinum derivate in BRCA2-mutated tumors, palliative chemotherapy with gemcitabine and oxaliplatin was initiated in November 2020. As with cisplatin, increasing polyneuropathy was reported by the patient. Furthermore, gemcitabine/oxaliplatin led to an aggravation of already existing pancytopenia due to portal hypertension and splenomegaly, the latter most likely resulting from medical-induced liver fibrosis evidenced by elevated liver stiffness in transient elastography (13 kPa) and postoperative stenosis of the portal vein. Eventually, after one cycle, chemotherapy had to be discontinued when platelet count decreased further under treatment.

In February 2020, an additional immunohistochemical examination of the recurrence lesion was conducted. In contrast to the initial biopsy of the primary lesion in 2017, immunohistochemistry revealed a positive PD-L1 status with positivity for PD-L1 in immune cells (CPS 30) and tumor cells (TPS 20) (see **Figure 3C**). Furthermore, the tumor tissue showed Her2neu overexpression with a FISH ratio ≥2. As an individual therapy concept and based on the molecular and histochemical tumor alterations, a switch to off-label second-line systemic therapy with a PARP inhibitor and a PD-1 inhibitor (pembrolizumab) was recommended by our interdisciplinary tumor conference in November 2020 for the patient, who still presented a great performance status (ECOG 0). Before therapy onset, written, informed consent was obtained from the patient. After 4 weeks of monotherapy with olaparib 300 mg twice daily, 200 mg of pembrolizumab was administered additionally every 3 weeks in combination with olaparib. Staging examinations were performed about every 3 months by CT of thorax and CT or MRI of the abdomen or MRI of the liver. First radiological imaging in January 2021 already showed no signs of CCA recurrence, consistent with complete remission (see **Figure 1F**). About 15 months later, up to April 2022, peritoneal manifestation and lymph node metastasis remained in complete remission without evidence of newly developed metastasis. The liver MRI also showed no new suspect intrahepatic lesions. Ca 125 decreased shortly after initiation of olaparib/pembrolizumab and has remained within normal range throughout since March 2021 (see **Figure 2**).

Olaparib was tolerated without any subjective side effects. However, with reoccurring pancytopenia and grade 3 anemia requiring transfusions of erythrocyte concentrates, the dosage of olaparib had to be modified to a 50% dose (150 mg twice daily). With this 50% dose adaptation, the therapy with olaparib was continuously tolerated by the patient.

Pembrolizumab was initially tolerated without side effects. In January 2021, TSH was noticeably suppressed (<0.01 µU/ml) for the first time, with elevated fT4 (2.9 ng/dl) and fT3 (5.84 pg/ml) as well as significantly increased antibodies against thyroglobulin (752 IU/ml) and TPO (356 IU/ml). Since the overall constellation of findings, including patient history with known Hashimoto’s thyroiditis, pointed toward an immune-related thyroiditis, we initiated thyreostatic therapy with carbimazol at 30 mg per day and referred the patient for endocrinological consultation. In line with typical findings of an acute autoimmune thyroiditis, sonographic thyroid examination showed an increased total volume of 45 ml and strong vascularization with only faint and diffuse tracer distribution of Tc-99-m pertechnetate in scintigraphy at the same time (see **Supplementary Figure 1**). Thyroid function normalized by April 2021. Sonographic examination confirmed a markedly decreased thyroid volume (10 ml) as well as reduced vascularization of the thyroid parenchyma. The patient has been closely monitored since and has maintained an euthyreotic metabolism until April 2022.

## Discussion

Here, we report on the efficacy, feasibility, and tolerability of a combination of a PARP inhibitor, olaparib, and a PD-1 inhibitor, pembrolizumab, as a second-line therapy after gemcitabine and platinum derivate in a patient with metastatic BRCA2-mutated and PD-L1-positive intrahepatic cholangiocarcinoma.

Cholangiocarcinoma is a comparably rare tumor entity with poor prognosis. When curative resection is not possible, palliative systemic chemotherapy remains as standard therapy. To date, most regimes introduce a gemcitabine- and platinum-based therapy as first line, based on the results of the ABC-02 study (20), followed by a 5-FU doublet combination as second-line (ABC-06) (21, 24). In recent years, tumor sequencing as part of the concept of personalized tumor therapy has emerged as a crucial tool.

Depending on the extra- or intrahepatic origin of the cholangiocarcinoma, different patterns of molecular alterations can be found in this disease (25). The most common genetic alterations of extrahepatic CCA include ARID1B, PRKACA, and PBRM compared to IDH, FGFR, BAP1, or PTEN in intrahepatic cholangiocarcinoma (7, 26). A few identified alterations have led to approved systemic therapy in second-line treatment. Ivosidenib, for example, has shown longer PFS in patients with IDH1 mutations (22). CCA with FGFR2 fusions are susceptible to pemigatinib (27). In a phase II basket trial (MyPathway) including pretreated biliary tract cancers with HER2neu amplification or overexpression, an objective response rate of 23% was reached with pertuzumab combined with trastuzumab (28). Of note, our patient showed immunohistochemical proof of Her2neu overexpression and a Her2neu/CEP17 FISH ratio greater than 2 without gene amplification of Her2 in next-generation sequencing. This finding is in line with previously published results that have described discordances between Her2 gene amplification and Her2neu expression (29, 30), e.g., related to chromosome 17 aberrations (31).

BRCA mutations, as evident in our patient, are a well-characterized target for PARP inhibitors (32). FDA and EMA approvals have been granted for ovarian cancer, breast cancer, prostate cancer, and pancreatic cancer. BRCA mutations in CCA are comparably rare (33).

There is only limited evidence from retrospective studies or case reports on PARP inhibitors in BRCA mutations in cholangiocarcinoma (34–37). However, these studies indicate a favorable response when treated with PARP inhibitors (34). Results of several phase II studies analyzing the effects of PARP inhibitors in CCA are pending (38).

Immune checkpoint inhibition (ICI) plays a growing role in a variety of tumor entities, partially even independent of PD-L1 status. Regarding CCA, the TOPAZ-1 phase III trial has recently shown a benefit for durvalumab (PD-L1 inhibition) combined with standard gemcitabine/cisplatin compared to chemotherapy alone (23). Further trials comparing the effects of ICI alone or in combination with chemotherapy, such as IMBRAVE 151 (atezolizumab plus gemcitabine/cisplatin), are ongoing (39). The Keynote-158 trial has, inter alia, analyzed the effects of pembrolizumab on PD-L1-positive and -negative CCA. Pembrolizumab monotherapy showed an ORR of 5.8% independent of PD-L1 status (40, 41). Combining ICI with PARP inhibitors could enhance the efficacy of both agents synergistically. Preclinical studies suggest that PARP inhibition induces tumor infiltration by immune cells *via* the STING pathway and leads to upregulation of PD-L1 expression, consequently enhancing susceptibility for ICI (42–45). PD-L1 blockade, on the other hand, counteracts the depletion of tumor-infiltrating lymphocytes, thereby potentiating the effects of PARP inhibitors in several *in vitro* and *in vivo* models (43, 45, 46). Furthermore, PARP inhibition is known to cause an accumulation of DNA damage which might in turn increase the mutation load of tumors. This effect could increase the expression of immune checkpoint receptors, such as PD-L1, on tumor cells increasing the predisposition to respond to checkpoint inhibition (47). A phase I trial by Friedland et al. demonstrated a response rate of 25% in advance solid cancer by combining ICI with a PARP inhibitor (48). Final results of an ongoing phase II trial studying the effects of pembrolizumab and olaparib are pending (49).

To our knowledge, the combination of ICI with a PARP inhibitor in cholangiocarcinoma has been described in only one further case (50). However, in the case described by Xiong et al., the combination with olaparib and pembrolizumab was used in a patient with BRCA1-mutated and PD-L1-positive CCA after recurrence following adoptive immunotherapy. A complete remission lasting for over 9 months was observed.

In contrast to the case described above, our patient presented with CCA with a BRCA-2 mutation, a PD-L1 CPS expression of 30%, and the fact that the patient had a previous exposition to platinum derivate.

Platinum-based chemotherapy has been shown to be effective in patients with BRCA-mutated tumors, mostly due to germline mutations. Recently, a sequential therapy with platinum derivate and the poly (ADP-ribose) polymerase inhibitor, olaparib as monotherapy, has shown clinical benefit in BRCA-mutated metastatic pancreatic cancer in the phase III trial (POLO), allowing approval of olaparib for these patients (34). Furthermore, platinum derivates can have immunomodulatory effects enhancing tumor immunity (51). Interestingly, in our case the patient was PD-L1 negative initially but showed significant PD-L1 expression after platinum induction. In preclinical and clinical observations, platinum treatment lead to an upregulation of PD-1/PD-L1 expression in tumor cells (52–54).

Although our patient also presented with HER2neu overexpression, which might respond to targeted therapy with pertuzumab and trastuzumab (28), we decided to switch to olaparib and pembrolizumab due to the prior platinum therapy and proof of BRCA2 mutation as well as positive PD-L1 status, especially as the patient did not progress under oxaliplatin therapy.

Already 12 weeks after therapy onset, the patient experienced a rapid response to the combined therapy resulting in an excellent tumor response with complete remission sustained for over 15 months. At the time of this report in April 2022, the patient was still in complete remission and presented an ECOG of 0. Immune-related thyroiditis and anemia were manageable adverse events and have not resulted in discontinuation of olaparib and pembrolizumab to this day. These results are supported by the results of numerous phase II trials on combination therapy of PARP inhibitors and ICI in breast and ovarian cancer (55, 56) but also in prostate cancer (57, 58) or basket trials including other solid tumors, such as gastric cancer (59). Most common adverse events were nausea and cytopenia. Occurrence of immune-related adverse events was comparable with monotherapy (44).

Preclinical data support the rationale of a superior effect of olaparib and pembrolizumab in our patient due to an induction with platinum derivate. Furthermore, the combination of checkpoint inhibition with PARP inhibition may induce a synergistic antitumoral effect. PARP inhibition is known to cause an accumulation of DNA damage, which in turn can increase the mutation load of tumors. This effect could increase the expression of immune checkpoint receptors, such as PD-L1, on tumor cells increasing the predisposition to respond to checkpoint inhibition (47).

In summary, the presented case showed substantial clinical activity of a combination therapy with olaparib and pembrolizumab in advanced BRCA2-mutated and PD-L1-positive cholangiocarcinoma as second-line therapy after platinum derivate chemotherapy. The therapy was well tolerated, but close screening for immune-related adverse events is necessary, especially in susceptible patient groups. Thus, identifying targetable molecular signatures and combinations of targeted therapies with immunotherapy revealed a promising strategy to effectively treat patients with cholangiocarcinoma and should be considered after failure of standard chemotherapy.

## Data availability statement

The original contributions presented in the study are included in the article/**supplementary material**. Further inquiries can be directed to the corresponding authors.

## Ethics statement

Written informed consent was obtained from the individual(s) for the publication of any potentially identifiable images or data included in this article.

## Author contributions

TZ: acquisition of data, drafting of the manuscript. RM: critical revision of the manuscript for important intellectual content. ChM: critical revision of the manuscript for important intellectual content. FS: critical revision of the manuscript for important intellectual content. CaM: figure curation, critical revision of the manuscript for important intellectual content. MT: figure curation, critical revision of the manuscript for important intellectual content. BSK: figure curation, critical revision of the manuscript for important intellectual content. ME: critical revision of the manuscript for important intellectual content. TG: critical revision of the manuscript for important intellectual content. HM: critical revision of the manuscript for important intellectual content. JK: critical revision of the manuscript for important intellectual content. CS: critical revision of the manuscript for important intellectual content. MG-C: acquisition of data, drafting of the manuscript. All authors contributed to the article and approved the submitted version.

## Funding

This work was supported by a BONFOR grant from the University of Bonn and grant number 109255 from the German Cancer Aid Association (Deutsche Krebshilfe) awarded to MG-C.

## Conflict of interest

M-GC has contributed to advisory boards for Roche, Eisai, MSD, BMS, AZ and Lilly.

The remaining authors declare that the research was conducted in the absence of any commercial or financial relationships that could be construed as a potential conflict of interest.

## Publisher’s note

All claims expressed in this article are solely those of the authors and do not necessarily represent those of their affiliated organizations, or those of the publisher, the editors and the reviewers. Any product that may be evaluated in this article, or claim that may be made by its manufacturer, is not guaranteed or endorsed by the publisher.

## Supplementary material

Supplementary Figure 1 **(A)** ultrasound examination of the thyroid gland from February 2021 shows an increased thyroid volume of about 45 ml (normal value in men: <25ml) as well as a strongly inhomogeneous, echo-rich internal structure without definable focal findings. Doppler sonography shows enhanced vascularization of the thyroid parenchyma. **(B)** Scintigraphy with 47MBq Tc-99 m pertechnetate from February 2021 displaying only faint, diffuse tracer enrichment. Tc-99m uptake: 0.31% of the administered activity.
