# Supplementary figures and images for: Case Report: Sustained complete remission on combination therapy with olaparib and pembrolizumab in BRCA2-mutated and PD-L1-positive metastatic cholangiocarcinoma after platinum derivate
Source: Front Oncol. 2022 Jul 25;12:933943. doi: 10.3389/fonc.2022.933943 (PMC9359099; doi:10.3389/fonc.2022.933943)

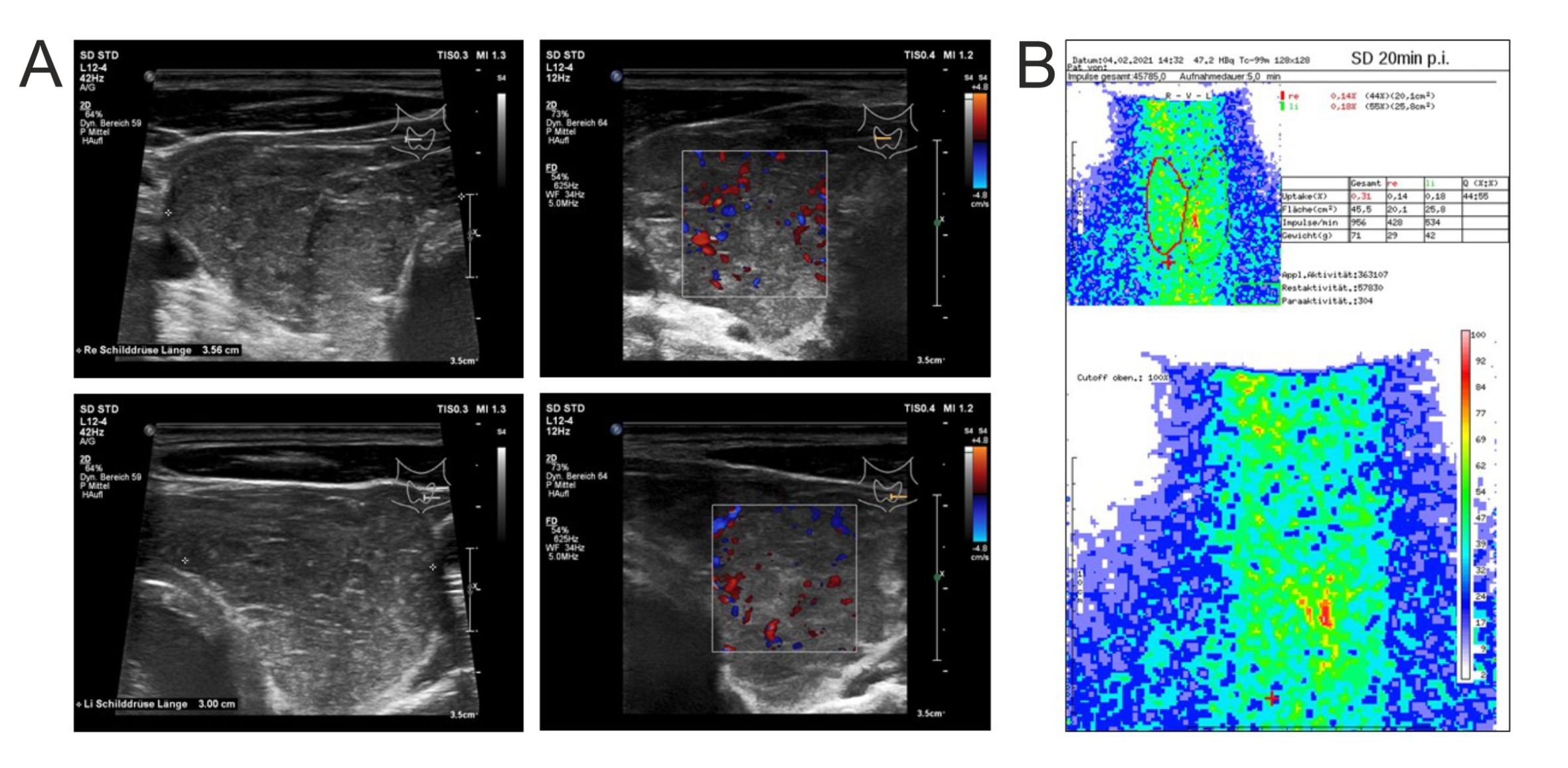

Supplement: Supplementary Figure 1 — (A) ultrasound examination of the thyroid gland from February 2021 shows an increased thyroid volume of about 45 ml (normal value in men: <25ml) as well as a strongly inhomogeneous, echo-rich internal structure without definable focal findings. Doppler sonography shows enhanced vascularization of the thyroid parenchyma. (B) Scintigraphy with 47MBq Tc-99 m pertechnetate from February 2021 displaying only faint, diffuse tracer enrichment. Tc-99m uptake: 0.31% of the administered activity. [file Image_1.tif]
